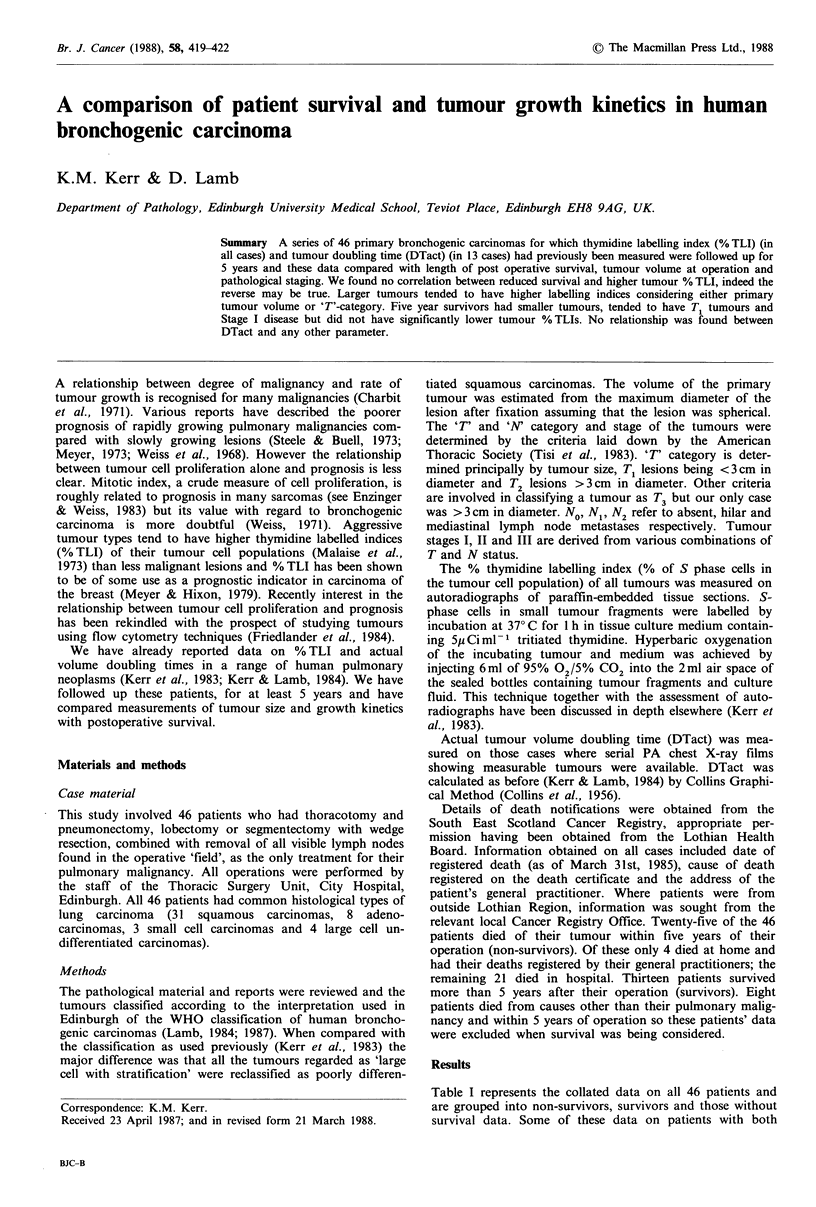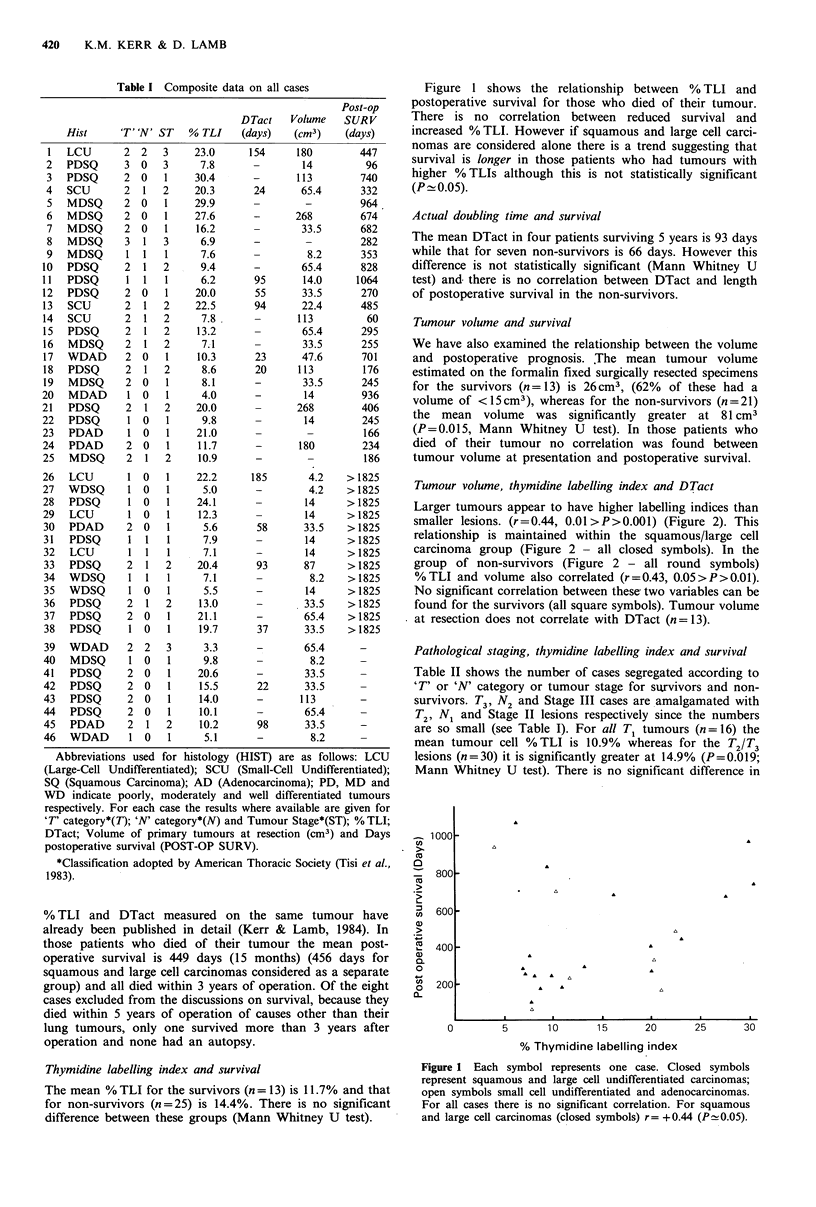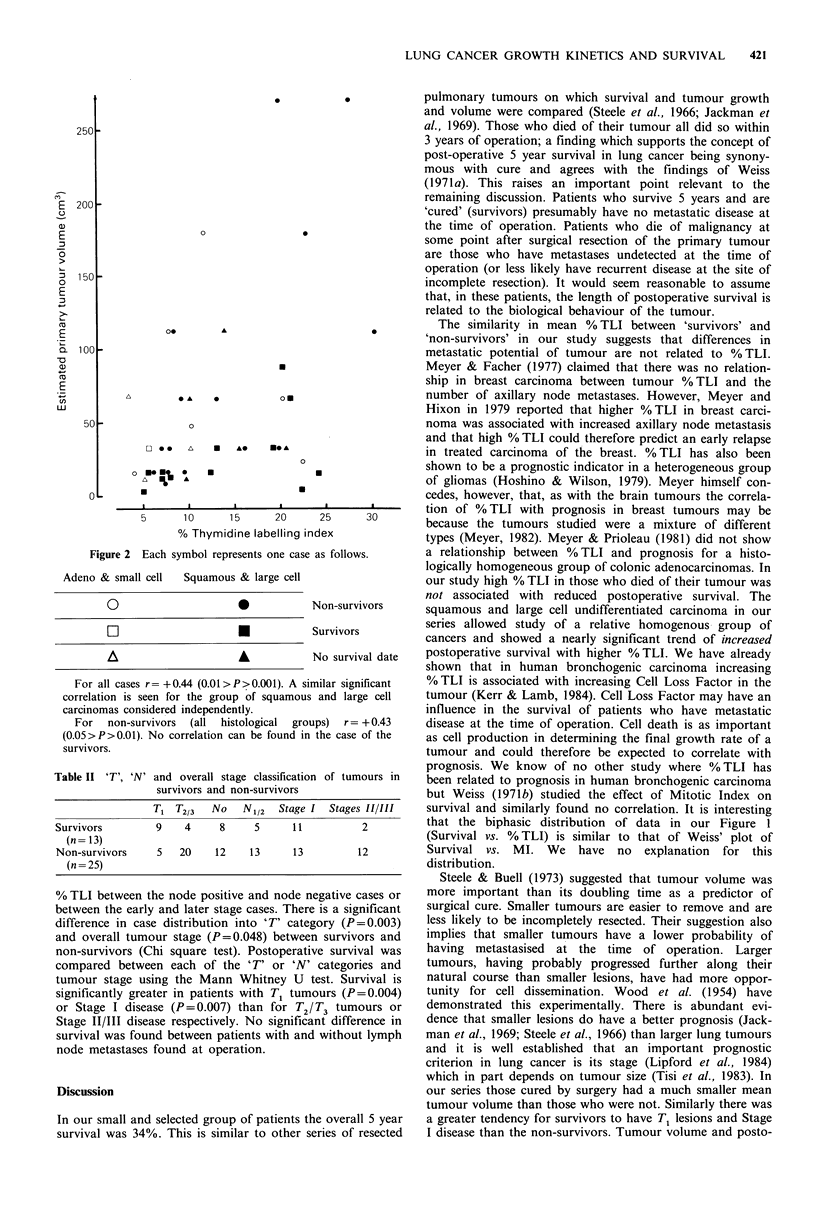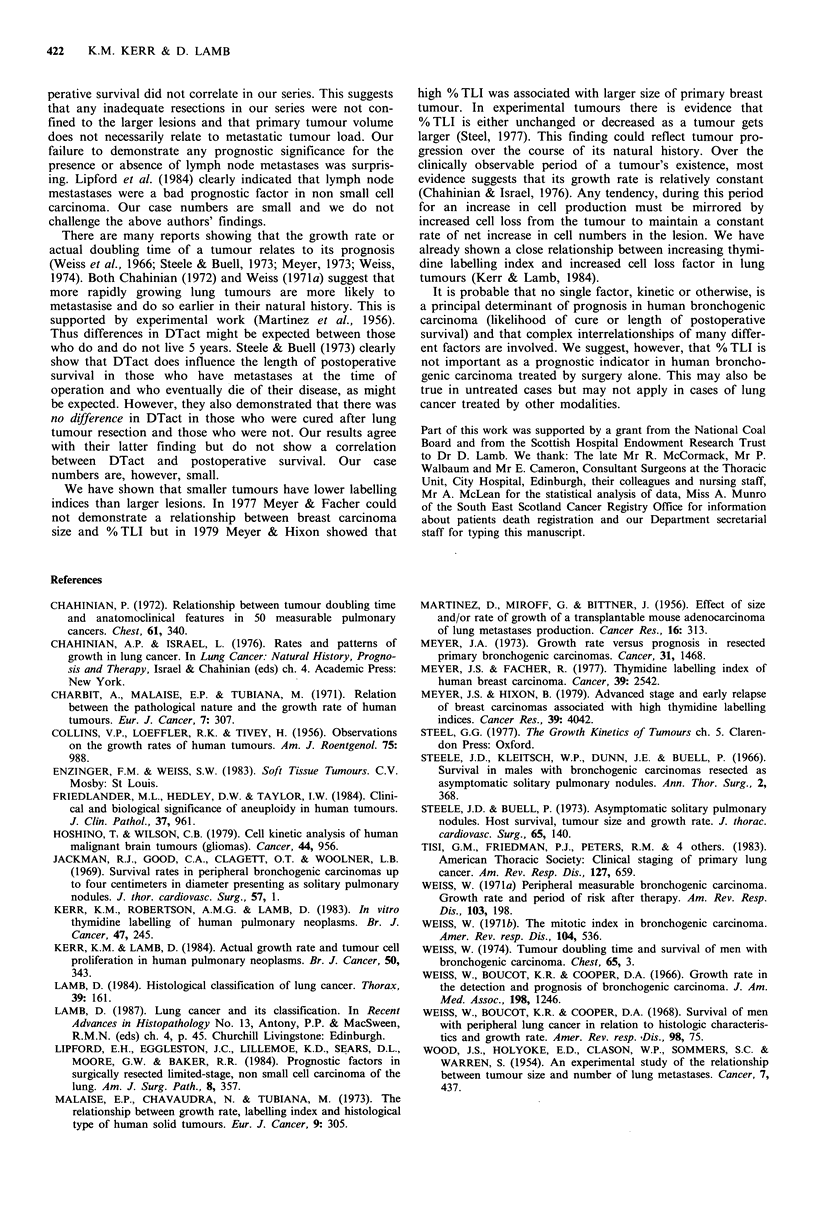# A comparison of patient survival and tumour growth kinetics in human bronchogenic carcinoma.

**DOI:** 10.1038/bjc.1988.233

**Published:** 1988-10

**Authors:** K. M. Kerr, D. Lamb

**Affiliations:** Department of Pathology, Edinburgh University Medical School, UK.

## Abstract

A series of 46 primary bronchogenic carcinomas for which thymidine labelling index (%TLI) (in all cases) and tumour doubling time (DTact) (in 13 cases) had previously been measured were followed up for 5 years and these data compared with length of post operative survival, tumour volume at operation and pathological staging. We found no correlation between reduced survival and higher tumour %TLI, indeed the reverse may be true. Larger tumours tended to have higher labelling indices considering either primary tumour volume or 'T'-category. Five year survivors had smaller tumours, tended to have T1 tumours and Stage I disease but did not have significantly lower tumour %TLIs. No relationship was found between DTact and any other parameter.


Br. J. Cancer (1988), 58, 419–422 © The Macmillan Press Ltd., 1988

### A comparison of patient survival and tumour growth kinetics in human bronchogenic carcinoma

K.M. Kerr & D. Lamb

*Department of Pathology, Edinburgh University Medical School, Teviot Place, Edinburgh EH8 9AG, UK.*

**Summary** A series of 46 primary bronchogenic carcinomas for which thymidine labelling index (%TLI) (in all cases) and tumour doubling time (DTact) (in 13 cases) had previously been measured were followed up for 5 years and these data compared with length of post operative survival, tumour volume at operation and pathological staging. We found no correlation between reduced survival and higher tumour %TLI, indeed the reverse may be true. Larger tumours tended to have higher labelling indices considering either primary tumour volume or '*T*'-category. Five year survivors had smaller tumours, tended to have $T_1$ tumours and Stage I disease but did not have significantly lower tumour %TLIs. No relationship was found between DTact and any other parameter.

A relationship between degree of malignancy and rate of tumour growth is recognised for many malignancies (Charbit *et al.*, 1971). Various reports have described the poorer prognosis of rapidly growing pulmonary malignancies compared with slowly growing lesions (Steele & Buell, 1973; Meyer, 1973; Weiss *et al.*, 1968). However the relationship between tumour cell proliferation alone and prognosis is less clear. Mitotic index, a crude measure of cell proliferation, is roughly related to prognosis in many sarcomas (see Enzinger & Weiss, 1983) but its value with regard to bronchogenic carcinoma is more doubtful (Weiss, 1971). Aggressive tumour types tend to have higher thymidine labelled indices (%TLI) of their tumour cell populations (Malaise *et al.*, 1973) than less malignant lesions and %TLI has been shown to be of some use as a prognostic indicator in carcinoma of the breast (Meyer & Hixon, 1979). Recently interest in the relationship between tumour cell proliferation and prognosis has been rekindled with the prospect of studying tumours using flow cytometry techniques (Friedlander *et al.*, 1984).

We have already reported data on %TLI and actual volume doubling times in a range of human pulmonary neoplasms (Kerr *et al.*, 1983; Kerr & Lamb, 1984). We have followed up these patients, for at least 5 years and have compared measurements of tumour size and growth kinetics with postoperative survival.

### Materials and methods

### *Case material*

This study involved 46 patients who had thoracotomy and pneumonectomy, lobectomy or segmentectomy with wedge resection, combined with removal of all visible lymph nodes found in the operative 'field', as the only treatment for their pulmonary malignancy. All operations were performed by the staff of the Thoracic Surgery Unit, City Hospital, Edinburgh. All 46 patients had common histological types of lung carcinoma (31 squamous carcinomas, 8 adenocarcinomas, 3 small cell carcinomas and 4 large cell undifferentiated carcinomas).

### *Methods*

The pathological material and reports were reviewed and the tumours classified according to the interpretation used in Edinburgh of the WHO classification of human bronchogenic carcinomas (Lamb, 1984; 1987). When compared with the classification as used previously (Kerr *et al.*, 1983) the major difference was that all the tumours regarded as 'large cell with stratification' were reclassified as poorly differentiated squamous carcinomas. The volume of the primary tumour was estimated from the maximum diameter of the lesion after fixation assuming that the lesion was spherical. The '*T*' and '*N*' category and stage of the tumours were determined by the criteria laid down by the American Thoracic Society (Tisi *et al.*, 1983). '*T*' category is determined principally by tumour size, $T_1$ lesions being $<3$ cm in diameter and $T_2$ lesions $>3$ cm in diameter. Other criteria are involved in classifying a tumour as $T_3$ but our only case was $>3$ cm in diameter. $N_0$, $N_1$, $N_2$ refer to absent, hilar and mediastinal lymph node metastases respectively. Tumour stages I, II and III are derived from various combinations of *T* and *N* status.

The % thymidine labelling index (% of *S* phase cells in the tumour cell population) of all tumours was measured on autoradiographs of paraffin-embedded tissue sections. *S*-phase cells in small tumour fragments were labelled by incubation at 37°C for 1 h in tissue culture medium containing $5\mu Ci\,ml^{-1}$ tritiated thymidine. Hyperbaric oxygenation of the incubating tumour and medium was achieved by injecting 6 ml of 95% $O_2$/5% $CO_2$ into the 2 ml air space of the sealed bottles containing tumour fragments and culture fluid. This technique together with the assessment of autoradiographs have been discussed in depth elsewhere (Kerr *et al.*, 1983).

Actual tumour volume doubling time (DTact) was measured on those cases where serial PA chest X-ray films showing measurable tumours were available. DTact was calculated as before (Kerr & Lamb, 1984) by Collins Graphical Method (Collins *et al.*, 1956).

Details of death notifications were obtained from the South East Scotland Cancer Registry, appropriate permission having been obtained from the Lothian Health Board. Information obtained on all cases included date of registered death (as of March 31st, 1985), cause of death registered on the death certificate and the address of the patient's general practitioner. Where patients were from outside Lothian Region, information was sought from the relevant local Cancer Registry Office. Twenty-five of the 46 patients died of their tumour within five years of their operation (non-survivors). Of these only 4 died at home and had their deaths registered by their general practitioners; the remaining 21 died in hospital. Thirteen patients survived more than 5 years after their operation (survivors). Eight patients died from causes other than their pulmonary malignancy and within 5 years of operation so these patients' data were excluded when survival was being considered.

### Results

Table I represents the collated data on all 46 patients and are grouped into non-survivors, survivors and those without survival data. Some of these data on patients with both

Correspondence: K.M. Kerr.
Received 23 April 1987; and in revised form 21 March 1988.

**Table I** Composite data on all cases

| | Hist | 'T' | 'N' | ST | % TLI | DTact (days) | Volume ($cm^3$) | Post-op SURV (days) |
|---|---|---|---|---|---|---|---|---|
| 1 | LCU | 2 | 2 | 3 | 23.0 | 154 | 180 | 447 |
| 2 | PDSQ | 3 | 0 | 3 | 7.8 | – | 14 | 96 |
| 3 | PDSQ | 2 | 0 | 1 | 30.4 | – | 113 | 740 |
| 4 | SCU | 2 | 1 | 2 | 20.3 | 24 | 65.4 | 332 |
| 5 | MDSQ | 2 | 0 | 1 | 29.9 | – | – | 964 |
| 6 | MDSQ | 2 | 0 | 1 | 27.6 | – | 268 | 674 |
| 7 | MDSQ | 2 | 0 | 1 | 16.2 | – | 33.5 | 682 |
| 8 | MDSQ | 3 | 1 | 3 | 6.9 | – | – | 282 |
| 9 | MDSQ | 1 | 1 | 1 | 7.6 | – | 8.2 | 353 |
| 10 | PDSQ | 2 | 1 | 2 | 9.4 | – | 65.4 | 828 |
| 11 | PDSQ | 1 | 1 | 1 | 6.2 | 95 | 14.0 | 1064 |
| 12 | PDSQ | 2 | 0 | 1 | 20.0 | 55 | 33.5 | 270 |
| 13 | SCU | 2 | 1 | 2 | 22.5 | 94 | 22.4 | 485 |
| 14 | SCU | 2 | 1 | 2 | 7.8 | – | 113 | 60 |
| 15 | PDSQ | 2 | 1 | 2 | 13.2 | – | 65.4 | 295 |
| 16 | MDSQ | 2 | 1 | 2 | 7.1 | – | 33.5 | 255 |
| 17 | WDAD | 2 | 0 | 1 | 10.3 | 23 | 47.6 | 701 |
| 18 | PDSQ | 2 | 1 | 2 | 8.6 | 20 | 113 | 176 |
| 19 | MDSQ | 2 | 0 | 1 | 8.1 | – | 33.5 | 245 |
| 20 | MDAD | 1 | 0 | 1 | 4.0 | – | 14 | 936 |
| 21 | PDSQ | 2 | 1 | 2 | 20.0 | – | 268 | 406 |
| 22 | PDSQ | 1 | 0 | 1 | 9.8 | – | 14 | 245 |
| 23 | PDAD | 1 | 0 | 1 | 21.0 | – | – | 166 |
| 24 | PDAD | 2 | 0 | 1 | 11.7 | – | 180 | 234 |
| 25 | MDSQ | 2 | 1 | 2 | 10.9 | – | – | 186 |
| 26 | LCU | 1 | 0 | 1 | 22.2 | 185 | 4.2 | >1825 |
| 27 | WDSQ | 1 | 0 | 1 | 5.0 | – | 4.2 | >1825 |
| 28 | PDSQ | 1 | 0 | 1 | 24.1 | – | 14 | >1825 |
| 29 | LCU | 1 | 0 | 1 | 12.3 | – | 14 | >1825 |
| 30 | PDAD | 2 | 0 | 1 | 5.6 | 58 | 33.5 | >1825 |
| 31 | PDSQ | 1 | 1 | 1 | 7.9 | – | 14 | >1825 |
| 32 | LCU | 1 | 1 | 1 | 7.1 | – | 14 | >1825 |
| 33 | PDSQ | 2 | 1 | 2 | 20.4 | 93 | 87 | >1825 |
| 34 | WDSQ | 1 | 1 | 1 | 7.1 | – | 8.2 | >1825 |
| 35 | WDSQ | 1 | 0 | 1 | 5.5 | – | 14 | >1825 |
| 36 | PDSQ | 2 | 1 | 2 | 13.0 | – | 33.5 | >1825 |
| 37 | PDSQ | 2 | 0 | 1 | 21.1 | – | 65.4 | >1825 |
| 38 | PDSQ | 1 | 0 | 1 | 19.7 | 37 | 33.5 | >1825 |
| 39 | WDAD | 2 | 2 | 3 | 3.3 | – | 65.4 | – |
| 40 | MDSQ | 1 | 0 | 1 | 9.8 | – | 8.2 | – |
| 41 | PDSQ | 2 | 0 | 1 | 20.6 | – | 33.5 | – |
| 42 | PDSQ | 2 | 0 | 1 | 15.5 | 22 | 33.5 | – |
| 43 | PDSQ | 2 | 0 | 1 | 14.0 | – | 113 | – |
| 44 | PDSQ | 2 | 0 | 1 | 10.1 | – | 65.4 | – |
| 45 | PDAD | 2 | 1 | 2 | 10.2 | 98 | 33.5 | – |
| 46 | WDAD | 1 | 0 | 1 | 5.1 | – | 8.2 | – |

Abbreviations used for histology (HIST) are as follows: LCU (Large-Cell Undifferentiated); SCU (Small-Cell Undifferentiated); SQ (Squamous Carcinoma); AD (Adenocarcinoma); PD, MD and WD indicate poorly, moderately and well differentiated tumours respectively. For each case the results where available are given for '*T*' category*(*T*); '*N*' category*(*N*) and Tumour Stage*(ST); %TLI; DTact; Volume of primary tumours at resection ($cm^3$) and Days postoperative survival (POST-OP SURV).

*Classification adopted by American Thoracic Society (Tisi *et al.*, 1983).

%TLI and DTact measured on the same tumour have already been published in detail (Kerr & Lamb, 1984). In those patients who died of their tumour the mean post-operative survival is 449 days (15 months) (456 days for squamous and large cell carcinomas considered as a separate group) and all died within 3 years of operation. Of the eight cases excluded from the discussions on survival, because they died within 5 years of operation of causes other than their lung tumours, only one survived more than 3 years after operation and none had an autopsy.

*Thymidine labelling index and survival*

The mean %TLI for the survivors ($n=13$) is 11.7% and that for non-survivors ($n=25$) is 14.4%. There is no significant difference between these groups (Mann Whitney U test).

Figure 1 shows the relationship between %TLI and postoperative survival for those who died of their tumour. There is no correlation between reduced survival and increased %TLI. However if squamous and large cell carcinomas are considered alone there is a trend suggesting that survival is *longer* in those patients who had tumours with higher %TLIs although this is not statistically significant ($P \simeq 0.05$).

*Actual doubling time and survival*

The mean DTact in four patients surviving 5 years is 93 days while that for seven non-survivors is 66 days. However this difference is not statistically significant (Mann Whitney U test) and there is no correlation between DTact and length of postoperative survival in the non-survivors.

*Tumour volume and survival*

We have also examined the relationship between the volume and postoperative prognosis. The mean tumour volume estimated on the formalin fixed surgically resected specimens for the survivors ($n=13$) is $26 cm^3$, (62% of these had a volume of $<15 cm^3$), whereas for the non-survivors ($n=21$) the mean volume was significantly greater at $81 cm^3$ ($P=0.015$, Mann Whitney U test). In those patients who died of their tumour no correlation was found between tumour volume at presentation and postoperative survival.

*Tumour volume, thymidine labelling index and DTact*

Larger tumours appear to have higher labelling indices than smaller lesions. ($r=0.44$, $0.01>P>0.001$) (Figure 2). This relationship is maintained within the squamous/large cell carcinoma group (Figure 2 – all closed symbols). In the group of non-survivors (Figure 2 – all round symbols) %TLI and volume also correlated ($r=0.43$, $0.05>P>0.01$). No significant correlation between these two variables can be found for the survivors (all square symbols). Tumour volume at resection does not correlate with DTact ($n=13$).

*Pathological staging, thymidine labelling index and survival*

Table II shows the number of cases segregated according to '*T*' or '*N*' category or tumour stage for survivors and non-survivors. $T_3$, $N_2$ and Stage III cases are amalgamated with $T_2$, $N_1$ and Stage II lesions respectively since the numbers are so small (see Table I). For *all* $T_1$ tumours ($n=16$) the mean tumour cell %TLI is 10.9% whereas for the $T_2/T_3$ lesions ($n=30$) it is significantly greater at 14.9% ($P=0.019$; Mann Whitney U test). There is no significant difference in

**Figure 1** Each symbol represents one case. Closed symbols represent squamous and large cell undifferentiated carcinomas; open symbols small cell undifferentiated and adenocarcinomas. For all cases there is no significant correlation. For squamous and large cell carcinomas (closed symbols) $r=+0.44$ ($P \simeq 0.05$).

**Figure 2** Each symbol represents one case as follows.

| Adeno & small cell | Squamous & large cell | |
|---|---|---|
| ○ | ● | Non-survivors |
| □ | ■ | Survivors |
| △ | ▲ | No survival date |

For all cases $r = +0.44$ ($0.01 > P > 0.001$). A similar significant correlation is seen for the group of squamous and large cell carcinomas considered independently.

For non-survivors (all histological groups) $r = +0.43$ ($0.05 > P > 0.01$). No correlation can be found in the case of the survivors.

**Table II** '*T*', '*N*' and overall stage classification of tumours in survivors and non-survivors

| | $T_1$ | $T_{2/3}$ | *No* | $N_{1/2}$ | *Stage I* | *Stages II/III* |
|---|---|---|---|---|---|---|
| Survivors ($n = 13$) | 9 | 4 | 8 | 5 | 11 | 2 |
| Non-survivors ($n = 25$) | 5 | 20 | 12 | 13 | 13 | 12 |

%TLI between the node positive and node negative cases or between the early and later stage cases. There is a significant difference in case distribution into '*T*' category ($P = 0.003$) and overall tumour stage ($P = 0.048$) between survivors and non-survivors (Chi square test). Postoperative survival was compared between each of the '*T*' or '*N*' categories and tumour stage using the Mann Whitney U test. Survival is significantly greater in patients with $T_1$ tumours ($P = 0.004$) or Stage I disease ($P = 0.007$) than for $T_2/T_3$ tumours or Stage II/III disease respectively. No significant difference in survival was found between patients with and without lymph node metastases found at operation.

### Discussion

In our small and selected group of patients the overall 5 year survival was 34%. This is similar to other series of resected pulmonary tumours on which survival and tumour growth and volume were compared (Steele *et al.*, 1966; Jackman *et al.*, 1969). Those who died of their tumour all did so within 3 years of operation; a finding which supports the concept of post-operative 5 year survival in lung cancer being synonymous with cure and agrees with the findings of Weiss (1971*a*). This raises an important point relevant to the remaining discussion. Patients who survive 5 years and are 'cured' (survivors) presumably have no metastatic disease at the time of operation. Patients who die of malignancy at some point after surgical resection of the primary tumour are those who have metastases undetected at the time of operation (or less likely have recurrent disease at the site of incomplete resection). It would seem reasonable to assume that, in these patients, the length of postoperative survival is related to the biological behaviour of the tumour.

The similarity in mean %TLI between 'survivors' and 'non-survivors' in our study suggests that differences in metastatic potential of tumour are not related to %TLI. Meyer & Facher (1977) claimed that there was no relationship in breast carcinoma between tumour %TLI and the number of axillary node metastases. However, Meyer and Hixon in 1979 reported that higher %TLI in breast carcinoma was associated with increased axillary node metastasis and that high %TLI could therefore predict an early relapse in treated carcinoma of the breast. %TLI has also been shown to be a prognostic indicator in a heterogeneous group of gliomas (Hoshino & Wilson, 1979). Meyer himself concedes, however, that, as with the brain tumours the correlation of %TLI with prognosis in breast tumours may be because the tumours studied were a mixture of different types (Meyer, 1982). Meyer & Prioleau (1981) did not show a relationship between %TLI and prognosis for a histologically homogeneous group of colonic adenocarcinomas. In our study high %TLI in those who died of their tumour was *not* associated with reduced postoperative survival. The squamous and large cell undifferentiated carcinoma in our series allowed study of a relative homogenous group of cancers and showed a nearly significant trend of *increased* postoperative survival with higher %TLI. We have already shown that in human bronchogenic carcinoma increasing %TLI is associated with increasing Cell Loss Factor in the tumour (Kerr & Lamb, 1984). Cell Loss Factor may have an influence in the survival of patients who have metastatic disease at the time of operation. Cell death is as important as cell production in determining the final growth rate of a tumour and could therefore be expected to correlate with prognosis. We know of no other study where %TLI has been related to prognosis in human bronchogenic carcinoma but Weiss (1971*b*) studied the effect of Mitotic Index on survival and similarly found no correlation. It is interesting that the biphasic distribution of data in our Figure 1 (Survival *vs.* %TLI) is similar to that of Weiss' plot of Survival *vs.* MI. We have no explanation for this distribution.

Steele & Buell (1973) suggested that tumour volume was more important than its doubling time as a predictor of surgical cure. Smaller tumours are easier to remove and are less likely to be incompletely resected. Their suggestion also implies that smaller tumours have a lower probability of having metastasised at the time of operation. Larger tumours, having probably progressed further along their natural course than smaller lesions, have had more opportunity for cell dissemination. Wood *et al.* (1954) have demonstrated this experimentally. There is abundant evidence that smaller lesions do have a better prognosis (Jackman *et al.*, 1969; Steele *et al.*, 1966) than larger lung tumours and it is well established that an important prognostic criterion in lung cancer is its stage (Lipford *et al.*, 1984) which in part depends on tumour size (Tisi *et al.*, 1983). In our series those cured by surgery had a much smaller mean tumour volume than those who were not. Similarly there was a greater tendency for survivors to have $T_1$ lesions and Stage I disease than the non-survivors. Tumour volume and postoperative survival did not correlate in our series. This suggests that any inadequate resections in our series were not confined to the larger lesions and that primary tumour volume does not necessarily relate to metastatic tumour load. Our failure to demonstrate any prognostic significance for the presence or absence of lymph node metastases was surprising. Lipford *et al.* (1984) clearly indicated that lymph node mestastases were a bad prognostic factor in non small cell carcinoma. Our case numbers are small and we do not challenge the above authors' findings.

There are many reports showing that the growth rate or actual doubling time of a tumour relates to its prognosis (Weiss *et al.*, 1966; Steele & Buell, 1973; Meyer, 1973; Weiss, 1974). Both Chahinian (1972) and Weiss (1971*a*) suggest that more rapidly growing lung tumours are more likely to metastasise and do so earlier in their natural history. This is supported by experimental work (Martinez *et al.*, 1956). Thus differences in DTact might be expected between those who do and do not live 5 years. Steele & Buell (1973) clearly show that DTact does influence the length of postoperative survival in those who have metastases at the time of operation and who eventually die of their disease, as might be expected. However, they also demonstrated that there was *no difference* in DTact in those who were cured after lung tumour resection and those who were not. Our results agree with their latter finding but do not show a correlation between DTact and postoperative survival. Our case numbers are, however, small.

We have shown that smaller tumours have lower labelling indices than larger lesions. In 1977 Meyer & Facher could not demonstrate a relationship between breast carcinoma size and %TLI but in 1979 Meyer & Hixon showed that high %TLI was associated with larger size of primary breast tumour. In experimental tumours there is evidence that %TLI is either unchanged or decreased as a tumour gets larger (Steel, 1977). This finding could reflect tumour progression over the course of its natural history. Over the clinically observable period of a tumour's existence, most evidence suggests that its growth rate is relatively constant (Chahinian & Israel, 1976). Any tendency, during this period for an increase in cell production must be mirrored by increased cell loss from the tumour to maintain a constant rate of net increase in cell numbers in the lesion. We have already shown a close relationship between increasing thymidine labelling index and increased cell loss factor in lung tumours (Kerr & Lamb, 1984).

It is probable that no single factor, kinetic or otherwise, is a principal determinant of prognosis in human bronchogenic carcinoma (likelihood of cure or length of postoperative survival) and that complex interrelationships of many different factors are involved. We suggest, however, that %TLI is not important as a prognostic indicator in human bronchogenic carcinoma treated by surgery alone. This may also be true in untreated cases but may not apply in cases of lung cancer treated by other modalities.

Part of this work was supported by a grant from the National Coal Board and from the Scottish Hospital Endowment Research Trust to Dr D. Lamb. We thank: The late Mr R. McCormack, Mr P. Walbaum and Mr E. Cameron, Consultant Surgeons at the Thoracic Unit, City Hospital, Edinburgh, their colleagues and nursing staff, Mr A. McLean for the statistical analysis of data, Miss A. Munro of the South East Scotland Cancer Registry Office for information about patients death registration and our Department secretarial staff for typing this manuscript.

### References

CHAHINIAN, P. (1972). Relationship between tumour doubling time and anatomoclinical features in 50 measurable pulmonary cancers. *Chest*, **61**, 340.

CHAHINIAN, A.P. & ISRAEL, L. (1976). Rates and patterns of growth in lung cancer. In *Lung Cancer: Natural History, Prognosis and Therapy*, Israel & Chahinian (eds) ch. 4. Academic Press: New York.

CHARBIT, A., MALAISE, E.P. & TUBIANA, M. (1971). Relation between the pathological nature and the growth rate of human tumours. *Eur. J. Cancer*, **7**: 307.

COLLINS, V.P., LOEFFLER, R.K. & TIVEY, H. (1956). Observations on the growth rates of human tumours. *Am. J. Roentgenol.* **75**: 988.

ENZINGER, F.M. & WEISS, S.W. (1983). *Soft Tissue Tumours*. C.V. Mosby: St Louis.

FRIEDLANDER, M.L., HEDLEY, D.W. & TAYLOR, I.W. (1984). Clinical and biological significance of aneuploidy in human tumours. *J. Clin. Pathol.*, **37**, 961.

HOSHINO, T. & WILSON, C.B. (1979). Cell kinetic analysis of human malignant brain tumours (gliomas). *Cancer*, **44**, 956.

JACKMAN, R.J., GOOD, C.A., CLAGETT, O.T. & WOOLNER, L.B. (1969). Survival rates in peripheral bronchogenic carcinomas up to four centimeters in diameter presenting as solitary pulmonary nodules. *J. thor. cardiovasc. Surg.*, **57**, 1.

KERR, K.M., ROBERTSON, A.M.G. & LAMB, D. (1983). *In vitro* thymidine labelling of human pulmonary neoplasms. *Br. J. Cancer*, **47**, 245.

KERR, K.M. & LAMB, D. (1984). Actual growth rate and tumour cell proliferation in human pulmonary neoplasms. *Br. J. Cancer*, **50**, 343.

LAMB, D. (1984). Histological classification of lung cancer. *Thorax*, **39**: 161.

LAMB, D. (1987). Lung cancer and its classification. In *Recent Advances in Histopathology* No. 13, Antony, P.P. & MacSween, R.M.N. (eds) ch. 4, p. 45. Churchill Livingstone: Edinburgh.

LIPFORD, E.H., EGGLESTON, J.C., LILLEMOE, K.D., SEARS, D.L., MOORE, G.W. & BAKER, R.R. (1984). Prognostic factors in surgically resected limited-stage, non small cell carcinoma of the lung. *Am. J. Surg. Path.*, **8**, 357.

MALAISE, E.P., CHAVAUDRA, N. & TUBIANA, M. (1973). The relationship between growth rate, labelling index and histological type of human solid tumours. *Eur. J. Cancer*, **9**: 305.

MARTINEZ, D., MIROFF, G. & BITTNER, J. (1956). Effect of size and/or rate of growth of a transplantable mouse adenocarcinoma of lung metastases production. *Cancer Res.*, **16**: 313.

MEYER, J.A. (1973). Growth rate versus prognosis in resected primary bronchogenic carcinomas. *Cancer*, **31**, 1468.

MEYER, J.S. & FACHER, R. (1977). Thymidine labelling index of human breast carcinoma. *Cancer*, **39**: 2542.

MEYER, J.S. & HIXON, B. (1979). Advanced stage and early relapse of breast carcinomas associated with high thymidine labelling indices. *Cancer Res.*, **39**: 4042.

STEEL, G.G. (1977). *The Growth Kinetics of Tumours* ch. 5. Clarendon Press: Oxford.

STEELE, J.D., KLEITSCH, W.P., DUNN, J.E. & BUELL, P. (1966). Survival in males with bronchogenic carcinomas resected as asymptomatic solitary pulmonary nodules. *Ann. Thor. Surg.*, **2**, 368.

STEELE, J.D. & BUELL, P. (1973). Asymptomatic solitary pulmonary nodules. Host survival, tumour size and growth rate. *J. thorac. cardiovasc. Surg.*, **65**, 140.

TISI, G.M., FRIEDMAN, P.J., PETERS, R.M. & 4 others. (1983). American Thoracic Society: Clinical staging of primary lung cancer. *Am. Rev. Resp. Dis.*, **127**, 659.

WEISS, W. (1971*a*) Peripheral measurable bronchogenic carcinoma. Growth rate and period of risk after therapy. *Am. Rev. Resp. Dis.*, **103**, 198.

WEISS, W. (1971*b*). The mitotic index in bronchogenic carcinoma. *Amer. Rev. resp. Dis.*, **104**, 536.

WEISS, W. (1974). Tumour doubling time and survival of men with bronchogenic carcinoma. *Chest*, **65**, 3.

WEISS, W., BOUCOT, K.R. & COOPER, D.A. (1966). Growth rate in the detection and prognosis of bronchogenic carcinoma. *J. Am. Med. Assoc.*, **198**, 1246.

WEISS, W., BOUCOT, K.R. & COOPER, D.A. (1968). Survival of men with peripheral lung cancer in relation to histologic characteristics and growth rate. *Amer. Rev. resp. Dis.*, **98**, 75.

WOOD, J.S., HOLYOKE, E.D., CLASON, W.P., SOMMERS, S.C. & WARREN, S. (1954). An experimental study of the relationship between tumour size and number of lung metastases. *Cancer*, **7**, 437.